# Exploring reasons for state-level variation in incidence of dialysis-requiring acute kidney injury (AKI-D) in the United States

**DOI:** 10.1186/s12882-020-02000-7

**Published:** 2020-08-10

**Authors:** Zijin Chen, Charles E. McCulloch, Neil R. Powe, Michael Heung, Rajiv Saran, Meda E. Pavkov, Nilka Rios Burrows, Raymond K. Hsu, Chi-yuan Hsu, for the Centers for Disease Control and Prevention Chronic Kidney Disease Surveillance Team Neil Powe, Tanushree Banerjee, Delphine Tuot, Chi-yuan Hsu, Charles McCulloch, Deidra Crews, Raymond Hsu, Vanessa Grubbs, Kirsten Bibbins-Domingo, Rajiv Saran, Zubin Modi, Debbie Gipson, Vahakn Shahinian, Brenda Gillespie, Hal Morgenstern, Michael Heung, William Herman, Jennifer Bragg-Gresham, Austin Stack, Rajesh Balkrishnan, Jerry Yee, Diane Steffick, Xiaosong Zhang, Jie Xiang, Yun Han, Maggie Yin, Kara Zivin, Emily Ginier, Vivian Kurtz, April Wyncott, Nilka Ríos Burrows, Mark Eberhardt, La Shaundra Everhart, Juanita Mondesire, Priti Patel, Meda Pavkov, Deborah Rolka, Sharon Saydah Larry Waller

**Affiliations:** 1 Department of Nephrology, Ruijin Hospital affiliated to Shanghai Jiao Tong University School of Medicine, Shanghai, China; 2 Division of Nephrology, Department of Medicine, University of California, San Francisco, San Francisco, CA USA; 3 Department of Epidemiology and Biostatistics, University of California, San Francisco, San Francisco, CA USA; 4 UCSF Center for Vulnerable Populations, Department of Medicine, University of California, San Francisco, California, USA; 5 Department of Medicine, San Francisco General Hospital, San Francisco, California, USA; 6 Division of Nephrology, Department of Medicine and Department of Epidemiology, University of Michigan, Ann Arbor, MI USA; 7 Kidney Epidemiology and Cost Center, University of Michigan, Ann Arbor, MI USA; 8 Division of Diabetes Translation, Centers for Disease Control and Prevention, Atlanta, GA USA

**Keywords:** Geographic variation, Chronic health condition, AKI-D, Ecological study

## Abstract

**Background:**

There is considerable state-level variation in the incidence of dialysis-requiring acute kidney injury (AKI-D). However, little is known about reasons for this geographic variation.

**Methods:**

National cross-sectional state-level ecological study based on State Inpatient Databases (SID) and the Behavioral Risk Factor Surveillance System (BRFSS) in 2011. We analyzed 18 states and six chronic health conditions (diabetes mellitus [diabetes], hypertension, chronic kidney disease [CKD], arteriosclerotic heart disease [ASHD], cancer (excluding skin cancer), and chronic obstructive pulmonary disease [COPD]). Associations between each of the chronic health conditions and AKI-D incidence was assessed using Pearson correlation and multiple regression adjusting for mean age, the proportion of males, and the proportion of non-Hispanic whites in each state.

**Results:**

The state-level AKI-D incidence ranged from 190 to 1139 per million population. State-level differences in rates of hospitalization with chronic health conditions (mostly < 3-fold difference in range) were larger than the state-level differences in prevalence for each chronic health condition (mostly < 2.5-fold difference in range). A significant correlation was shown between AKI-D incidence and prevalence of diabetes, ASHD, and COPD, as well as between AKI-D incidence and rate of hospitalization with hypertension. In regression models, after adjusting for age, sex, and race, AKI-D incidence was associated with prevalence of and rates of hospitalization with five chronic health conditions--diabetes, hypertension, CKD, ASHD and COPD--and rates of hospitalization with cancer.

**Conclusions:**

Results from this ecological analysis suggest that state-level variation in AKI-D incidence may be influenced by state-level variations in prevalence of and rates of hospitalization with several chronic health conditions. For most of the explored chronic conditions, AKI-D correlated stronger with rates of hospitalizations with the health conditions rather than with their prevalences, suggesting that better disease management strategies that prevent hospitalizations may translate into lower incidence of AKI-D.

## Background

Dialysis-requiring acute kidney injury (AKI-D) is an important cause of morbidity and mortality in hospitalized patients and is associated with a large public health burden. We have recently reported considerable geographic variation in incidence of AKI-D in the United States [1–3] with some states having more than ten-fold higher incidence than others. However, little is known about reasons for this geographic variation.

We hypothesized that geographic variation in prevalence of certain chronic health conditions predisposing either directly or indirectly to acute kidney injury may be an important contributor to the observed geographic variation in AKI-D incidence. Candidate chronic conditions include diabetes mellitus (diabetes), hypertension, chronic kidney disease (CKD), arteriosclerotic heart disease (ASHD), cancer, and chronic obstructive pulmonary disease (COPD).

Furthermore, since AKI-D occurs only among hospitalized patients (since acute dialysis cannot be initiated in the outpatient setting), we hypothesized that geographic variation in the hospitalization rates of patients with the chronic health conditions is also potentially important.

To explore these associations, we analyzed data from 18 states derived from two community representative US databases—the State Inpatient Databases (SID) [4] and the Behavioral Risk Factor Surveillance System (BRFSS) [5].

## Methods

### Study design

We conducted a national, cross-sectional, ecological study among populations aged 45 years or older in the United States. We limited our study to adults aged 45 years or older because AKI-D incidence and chronic health conditions prevalence and hospitalizations are low in younger populations.

### Determining state-level variations in prevalence of chronic health conditions using BRFSS

Based on the existing literature, we selected a priori which chronic health conditions to study as candidates to correlate with AKI-D incidence. We included the top causes of ESRD (which we presume to be top causes of CKD also), the top morbid conditions accompanying of ESRD (as they may act as triggers of acute on chronic kidney disease), and possible risk factors for AKI-D from previous study [2, 6, 7]. Specifically, we first determined the top four primary causes of end-stage renal disease (ESRD) from the United States Renal Data System (USRDS): diabetes, hypertension, glomerulonephritis, and cystic kidney disease [6]. We then determined the top ten comorbid conditions reported among ESRD patients from the USRDS: hypertension, diabetes, chronic heart failure, arteriosclerotic heart disease, other cardiac disease, peripheral vascular disease, COPD, cerebrovascular accident/transient ischemic attack (CVA/TIA), diabetic retinopathy, and malignant neoplasm [7]. We finally noted the top ten diagnosis codes related to temporal trends in AKI-D from a prior study: shock, cardiac arrest and ventricular fibrillation, septicemia, multiple myeloma, respiratory failure, coagulation and hemorrhagic disorders, hypertension, coma stupor and brain damage, liver disease, and mycoses [2].

State-level prevalence estimates of chronic health conditions were obtained based on self-report data from the BRFSS in 2011. The BRFSS is the nation’s premier system of health-related telephone surveys that collect data at the state and local levels from US residents regarding their health-related risk behaviors, chronic health conditions, and use of preventive services [5, 8]. These data come from participants in all 50. Starting in 2011, a cell phone survey and a new methodology to calculate weighted values were included as components of the BRFSS [9, 10]. Because the 2011 BRFSS survey did not capture all the health conditions listed above, we consolidated primary causes of ESRD, the top ten comorbid conditions among ESRD patients, and the top ten diagnosis codes related to temporal trends of AKI-D into six chronic health conditions: diabetes, hypertension, CKD, ASHD, cancer (excluding skin cancer) (henceforth referred to simply as “cancer”), and COPD. The selection of these six health conditions was made prior to performing any association analyses.

The BRFSS questions and answer choices for these six chronic health conditions are listed in Supplementary Table 1 [11]. When the answer for each question was “yes”, we considered the participant to have this chronic health condition. When the answer was “no,” we considered the participant to not have this chronic health condition. For ASHD, two relevant questions (concerning “myocardial infarction” and “angina or coronary heart disease”) were included and ASHD classification was based on answering yes to either. Answers given as “missing,” “refused” or “don’t know/not sure” together accounted for < 3% of responses for all conditions and were classified as “no.”

### Determining state-level variations in incidence of hospitalized AKI-D using SID and US census

We used the State Inpatient Databases (SID) to determine the number of AKI-D hospitalizations [3, 4]. We then used US Census Bureau data to calculate state-by-state incidence of AKI-D.

AKI-D was defined when there was both an acute renal failure diagnostic code (International Classification of Diseases, Ninth Revision, Clinical Modification [ICD-9] codes 584.5, 584.6, 584.7, 584.8, or 584.9) and a dialysis procedure code (39.95, V45.11, V45.12, V56.0, or V56.2) without concurrent arteriovenous fistula creation or revision procedure codes (39.27, 39.42, 39.43, or 39.93) [1, 3, 12, 13]. Prior studies have showed that this algorithm produced ≥90% sensitivity, specific, positive and negative predictive values [1, 3, 12, 13]..

Due to cost reasons, we purchased only 25 out of the 30 SID available in 2011: Arizona (AZ), Arkansas (AR), California (CA), Colorado (CO), Florida (FL), Iowa (IA), Kentucky (KY), Massachusetts (MA), Maryland (MD), Maine (ME), Michigan (MI), Mississippi (MS), North Carolina (NC), Nebraska (NE), New Jersey (NJ), New Mexico (NM), New York (NY), Nevada (NE), Oregon (OR), Rhode Island (RI), South Carolina (SC), Utah (UT), Vermont (VT), Washington (WA), and West Virginia (WV) (Supplementary Figure 1) [3].

Nineteen SID databased reported > 15 diagnostic codes. In none of these 19 states did more than a quarter of the hospitalizations have > 15 diagnostic codes. 19 (non-overlapping) SID databases reported > 6 procedure codes. In none of these 19 states did more than a tenth of the hospitalizations have > 6 procedure codes [3]

In our primary analysis, to reduce ascertainment bias, we did not include SID databases with fewer than 15 diagnostic codes (ME, NE) and we only analyzed the first 15 diagnostic codes listed for each hospitalization (Supplementary Figure 1). We also only analyzed the first 6 procedure codes in our primary analysis (all states had at least this number of procedure codes) [3].

We counted AKI-D hospitalizations with diagnosis of ESRD (585.6) on discharge but not on admission. Our primary analysis did not include states (CO, MS, NC, UT, WV) in which we could not tell whether ESRD was an admission diagnosis or a discharge diagnosis [or both]) (Supplementary Figure 1) [3].

Our primary analysis was therefore based on 18 states (AZ, AR, CA, FL, IA, KY, MA, MD, MI, NJ, NM, NY, NV, OR, RI, SC, VT, WA) (Supplementary Figure 1) [3].

We used data from all 25 states in sensitivity analyses. Here, we only analyzed up to 9 diagnostic codes and up to 6 procedure codes for each state to reduce ascertainment bias (all states reported at least 9 diagnostic codes and 6 procedure codes) and excluded all hospitalizations containing a diagnostic code for ESRD (585.6) since for five states (CO, MS, NC, UT, and WV), we could not tell whether a this diagnosis was already present on admission [3].

### Determining state-level variations in rates of hospitalization with each of the chronic health conditions from SID, BRFSS, and US census

We identified and compiled hospital discharges related to each chronic health condition and calculated rates using estimated state-level patient populations. We hypothesized that hospitalization rate with each chronic health condition could be influenced driven by patients with that chronic health condition who had more severe disease or had complications that needed to be treated in the hospital.

Specifically, we used Clinical Classifications Software (CCS) in SID which collapses the > 14,000 standardized diagnosis codes in the International Classification of Diseases-Ninth Revision-Clinical Modification (ICD-9-CM) into a smaller number of clinically significant categories [3]. Each one of the six chronic health conditions of interest were mapped to the following CCS diagnostic codes: diabetes (CCS diagnostic code 49, 50), hypertension (CCS diagnostic code 98, 99), CKD (CCS diagnostic code 156, 158), ASHD (CCS diagnostic code 100, 101, 102), cancer (CCS diagnostic code 11–21 and 24–43), and COPD (CCS diagnostic code 127).

### Statistical methods

State-level AKI-D incidences were expressed as per million population (pmp) as described previously [3, 12, 14]. We calculated fold-difference for this by dividing the highest observed state-level AKI-D incidence by the lowest observed state-level AKI-D incidence. We classify states into low/medium/high tiers of AKI-D incidence to show regional variations on a map of the U.S..

We computed the chronic condition prevalence estimates from the BRFSS and the resident population from the 2011 US Census Bureau [15]. We calculated rate of hospitalizations with each chronic health condition per 1000 population as: count of hospitalizations / (chronic condition prevalence * resident population) * 1000. Fold-differences for these were also calculated taking highest vs. lowest observed values. (All incident rates are for per annum.)

To obtain prevalence estimates and associated confidence intervals (CIs), we used complex sample survey design specifying strata, clusters and sample weights for the combined landline telephone and cellular telephone data (“STSTR” was used for strata, “_PSU” for clusters, and “_LLCPWT” for sampling weights) [16].

Pearson correlation coefficients were used to determine i) the state-level correlation between prevalence of each chronic health condition and AKI-D incidence, and ii) the state-level correlation between rate of hospitalization with each chronic health condition and AKI-D incidence.

Linear regression analysis was performed to determine the state-level association between the prevalence of or rate of hospitalization with each chronic health condition and AKI-D incidence, adjusting for mean age, the proportion of males, and the proportion of non-Hispanic whites in each state. Size of parameter estimates and *p*-values from the linear regression models were used to gauge which chronic health condition may have had the greatest influence on AKI-D incidence. We also compared adjusted R-squared values in different models for each chronic health condition to evaluate whether differences in prevalence or rate of hospitalization with some chronic health conditions could potentially account for more of the observed state-level variation in AKI-D incidence.

In all regression models, we checked the linearity assumption. If the relationship between an exposure of interest and the outcome appeared non-linear, we added a quadratic term into the model and displayed our results graphically.

As a negative control, we examined skin cancer as a chronic health condition that we hypothesized would not have any correlation or association with AKI-D incidence. Information on skin cancer was also based on self-report in BRFSS (Supplementary Table 1) and we used CCS diagnostic code 22, 23 in SID.

Data were analyzed using STATA version 14.1 (Stata Corp., College Station, TX) and verified independently by a separate analyst using SAS version 9.4 (SAS Institute Inc., Cary, NC).

## Results

Demographic characteristics of BRFSS respondents and patients who had AKI-D hospitalizations are shown in Table 1**(**state level breakdown in Supplementary Table 2 and Supplementary Table 3). Individuals with AKI-D hospitalizations were more likely to be older, male, and non-Hispanic Black than BRFSS respondents.

**Table 1:** Demographic characteristics of AKI-D hospitalizations and the BRFSS population in patients aged 45 years or older for the 18 states in the primary analysis

	AKI-D Hospitalizations (*N* = 34,122)	BRFSS Population (weighted *N* = 57,396,000)
Mean age (SD), yrs	68.2 (12.0)^a^	61.1 (0.04)
Male, %	57.8	46.9
Race		
Non-Hispanic White, %	68.4	69.7
Non-Hispanic Black, %	14.3	8.7
Hispanic, %	11.1	12.9

^a^Excluding one state (SC) which only reported age categories

### Incidence of hospitalized AKI-D

**Fig. 1:**
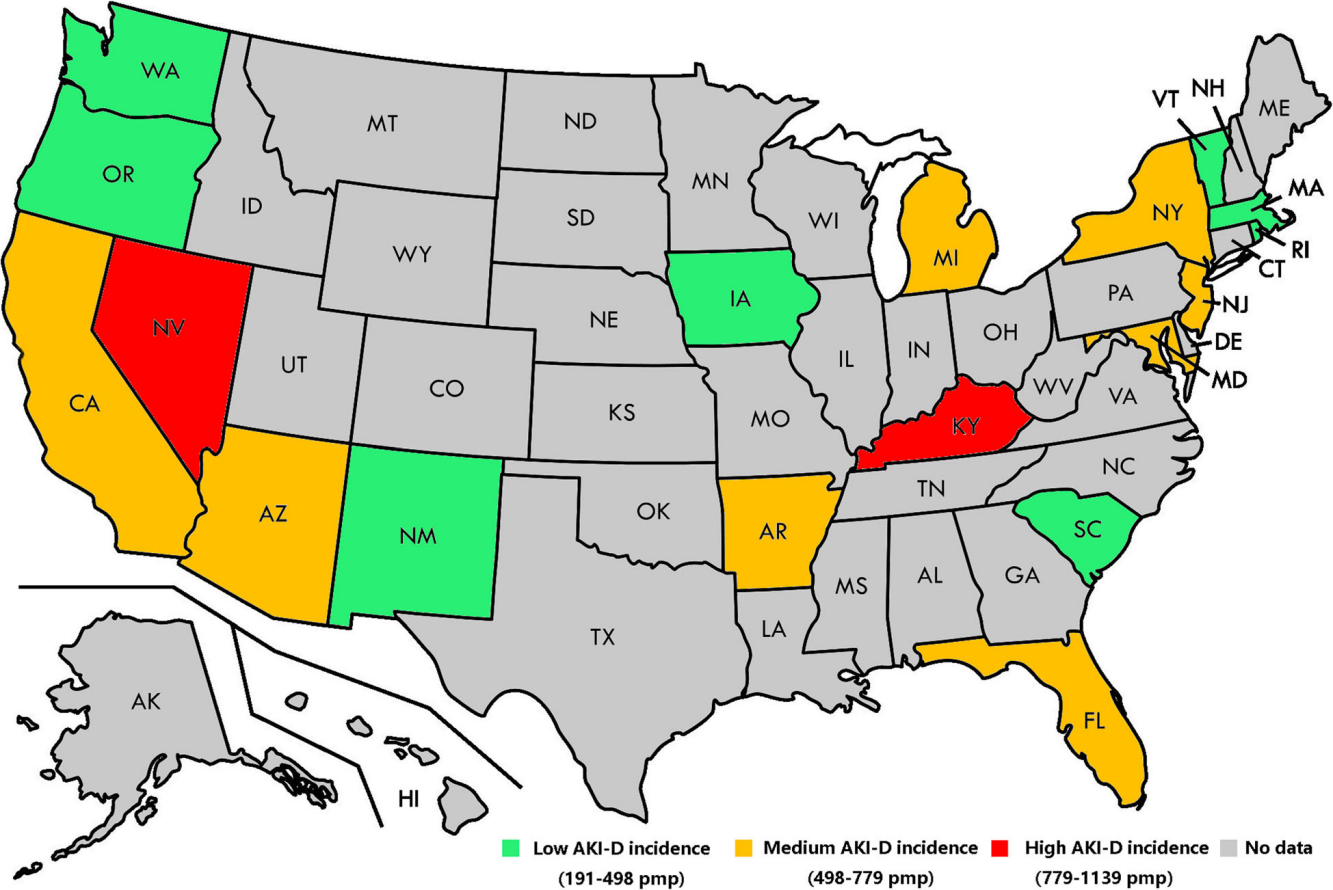
Map demonstrating the regional variation of AKI-D incidence in primary analysis with 18 states. The map was drawn using Adobe Photoshop CS6 version 13.1.2 URL:http://www.adobe.com

We identified 34,122 hospitalizations with AKI-D in SID of 18 states. The state-level AKI-D incidence varied considerably (Figure 1), ranging from 190 to 1139 pmp (6.0-fold difference) (Supplementary Table 2).

### Prevalence of chronic health conditions

The study included 188,997 participants from the BRFSS among individuals aged 45 years or older in 18 states (weighted population size = 57,396,000). Estimated prevalence of the six chronic health conditions of interest varied from state to state (Supplementary Tables 4–9). For diabetes, the range of state-level prevalence was 11.9 to 18.4% (1.5-fold difference), for hypertension 42.1 to 54.1% (1.3-fold difference), for CKD 2.4 to 5.9% (2.5-fold difference), for ASHD 9.3 to 15.8% (1.7-fold difference), for cancer 9.1 to 11.9% (1.3-fold difference), and for COPD 6.3 to 13.5% (2.1-fold difference).

### Rates of hospitalization rates with each of the chronic health conditions

Compared with state-level variations in disease prevalence, there was greater state-to-state variation in the rate hospitalization with the chronic health conditions (e.g., comparing fold-difference from highest to lowest state). Specifically, rates of hospitalization with diabetes ranged from 252 to 438 per 1000 patients (1.7-fold difference), whereas that with hypertension ranged from 153 to 278 per 1000 patients (1.8-fold difference), that with CKD 360 to 1144 per 1000 patients (3.2-fold difference), that with ASHD 289 to 547 per 1000 patients (1.9-fold difference), that with cancer from 197 to 384 per 1000 patients (1.9-fold difference), and that with COPD from 213 to 473 per 1000 patients (2.2-fold difference).

Note the 6-fold range difference in AKI-D incidence among states (1139/190 pmp) is more than the state-level variation in chronic health condition prevalence (mostly < 2.5-fold difference in range) or state-level variation in chronic health condition hospitalization rate (mostly < 3-fold difference in range).

### State-level correlation between prevalence of each chronic health condition and AKI-D incidence

**Fig. 2:**
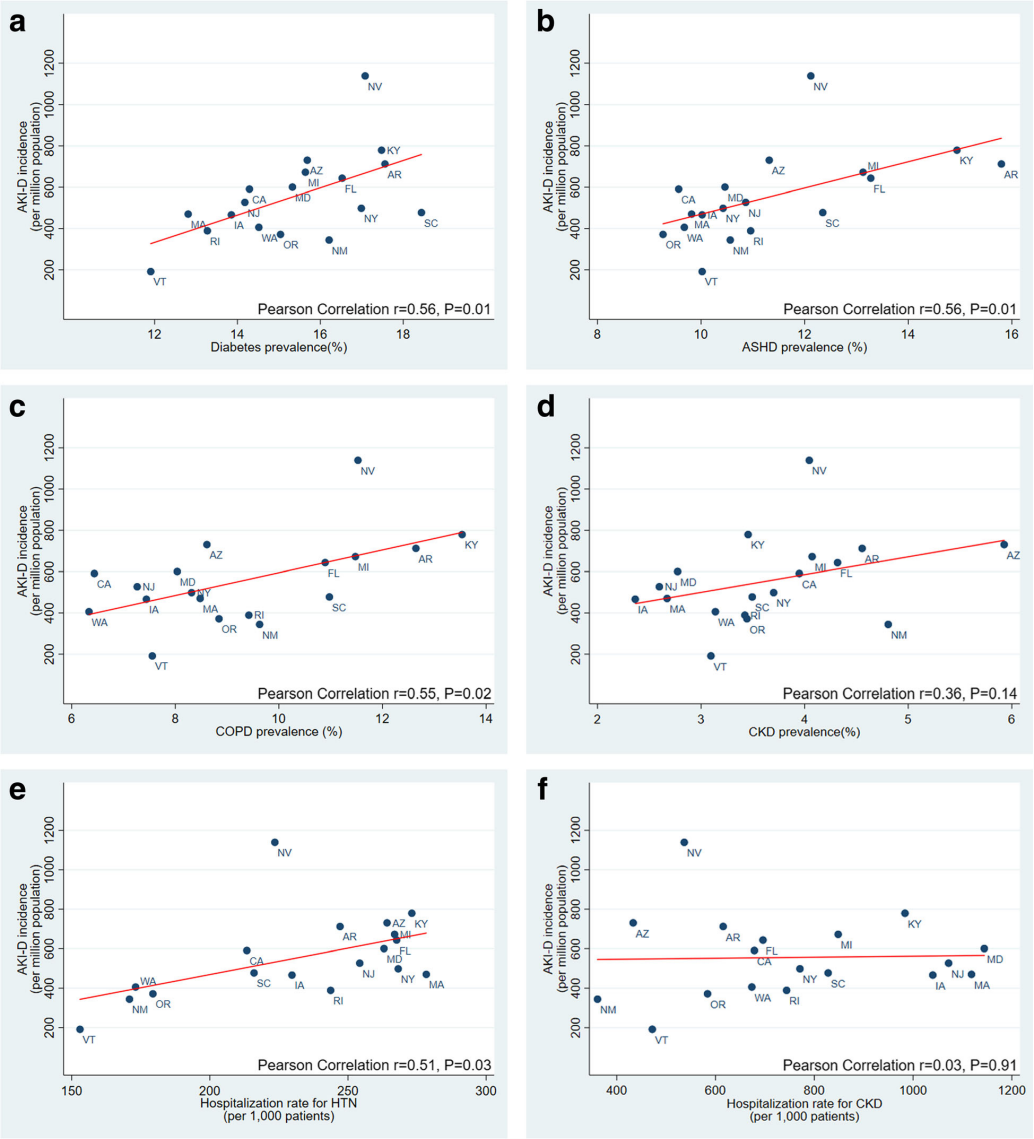
Pearson correlation between AKI-D incidence and selected chronic health conditions for the 18 states in the primary analysis. **a** AKI-D incidence and diabetes prevalence. **b** AKI-D incidence and ASHD prevalence. **c** AKI-D incidence and COPD prevalence. **d** AKI-D incidence and CKD prevalence. **e** AKI-D incidence and rate of hospitalization with hypertension. **f** AKI-D incidence and rate of hospitalization with CKD

At the state-level, a significant correlation was shown between AKI-D incidence and prevalence of diabetes (r = 0.56; *p* = 0.01, Fig. 2a), ASHD (r = 0.56; *p* = 0.01, Fig. 2b), and COPD (r = 0.55; *p* = 0.02, Fig. 2c). No significant correlation at the state-level was noted between AKI-D incidence and prevalence of hypertension (r = 0.35; *p* = 0.15), CKD (r = 0.36; *p* = 0.14, Fig. 2d) and cancer (r = 0.06; *p* = 0.82).

### State-level correlation between rate of hospitalization with each chronic health condition and AKI-D incidence

A significant correlation was also shown between AKI-D incidence and rate of hospitalization with hypertension (r = 0.51; *p* = 0.03, Fig. 2e). No significant correlation was shown for rates of hospitalization with diabetes (r = 0.27; *p* = 0.27), CKD (r = 0.03; *p* = 0.91, Fig. 2f), ASHD (r = 0.17; *p* = 0.49), cancer (r = 0.23; *p* = 0.37), and COPD (r = 0.13; *p* = 0.60).

### Association between the prevalence of and/or rates of hospitalization rate with each chronic health condition and AKI-D incidence by linear regression

After controlling for state-level differences in age, sex, and proportion of non-Hispanic whites, states with higher prevalence of diabetes, hypertension, ASHD, and COPD had higher incidence of AKI-D (Table 2). For example, for each 1% higher prevalence of diabetes in the state, we estimated there was a 70.5 (95% CI: 14.6–126.3) pmp higher incidence of AKI-D in the state.

**Table 2:** Linear regression model for AKI-D incidence and each chronic health condition for the 18 states in the primary analysis

	Prevalence (%)		Hospitalization rate (per 1000 patients)		
	Coef. (95% CI)	*P* value	Coef. (95% CI)	*P* value	Adjusted R^2^
*Model 1*					
Diabetes mellitus	70.5 (14.6126.3)	0.02	–	–	0.350
Hypertension	34.6 (10.5,58.7)	0.01	–	–	0.414
CKD	65.0 (−86.8216.8)	0.37	–	–	0.345
ASHD	70.4 (31.8108.9)	< 0.01	–	–	0.661
Cancer (excluding skin cancer)	−41.4 (− 204.7121.9)	0.59	–	–	0.266
COPD	54.6 (18.1,91.1)	< 0.01	–	–	0.600
Skin Cancer	28.1 (−58.7,11.5)	0.49	–	–	0.324
*Model 2*					
Diabetes mellitus	–	–	3.0 (1.8,4.1)	< 0.01	0.780
Hypertension	–	–	4.5 (2.8,6.1)	< 0.01	0.720
CKD	–	–	0.6 (0.0,1.1)	0.05	0.251
ASHD	–	–	2.3 (1.0,3.6)	< 0.01	0.509
Cancer (excluding skin cancer)	–	–	See Fig. 3a	< 0.01	0.648
COPD	–	–	1.7 (0.3,3.1)	0.02	0.573
Skin Cancer	–	–	4.1 (−16.4,24.6)	0.67	0.305
*Model 3*					
Diabetes mellitus	53.4 (42.7,64.1)	< 0.01	2.9 (2.6,3.3)	< 0.01	0.980
Hypertension	25.6 (17.7,33.5)	< 0.01	3.9 (3.1,4.7)	< 0.01	0.940
CKD	237.7 (143.9331.6)	< 0.01	1.0 (0.6,1.3)	< 0.01	0.823
ASHD	61.7 (41.9,81.4)	< 0.01	1.6 (1.0,2.2)	< 0.01	0.915
Cancer (excluding skin cancer)	23.9 (− 79.8127.6)	0.62	See Fig. 3b	< 0.01	0.625
COPD	55.7 (37.4,74.1)	< 0.01	1.9 (1.2,2.5)	< 0.01	0.916
Skin Cancer	48.1 (36.6132.9)	0.24	9.4 (−10.2,28.9)	0.31	0.296

Model 1: adjusted for mean age, % male, % non-Hispanic White, prevalence of each chronic health condition

Model 2: adjusted for mean age, % male, % non-Hispanic White, hospitalization rate of each chronic health condition

Model 3: adjusted for mean age, % male, % non-Hispanic White, prevalence of each chronic health condition and hospitalization rate of each chronic health condition

Abbreviations: *ASHD* Arteriosclerotic heart disease, *CKD* Chronic kidney disease, *COPD* Chronic obstructive pulmonary disease

**Fig. 3:**
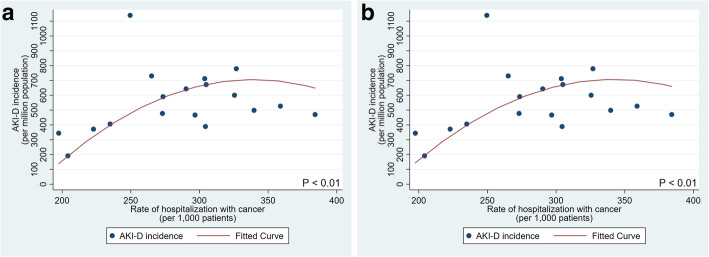
Rate of hospitalization with cancer and predicted AKI-D incidence for the 18 states in the primary analysis. **a** Adjusted for age, % male, and % non-Hispanic White. **b** Adjusted for age, % male, % non-Hispanic White, and prevalence of cancer

We also found that states with higher rates of hospitalization with any of the selected six chronic health conditions had higher incidence of AKI-D (with CKD being borderline/ having the weakest association). Generally, these relationships were adequately captured in a linear model. For example, for each 1 per 1000 higher rate of hospitalization with diabetes, we estimated there was a 3.0 (95% CI: 1.8–4.1) pmp higher incidence of AKI-D in the state. For cancer, states with higher hospitalization rates also had higher AKI-D incidence but the association was not linear and is more accurately shown graphically in Fig. 3a.

We observed that for most of the chronic conditions, models with hospitalization rate as the main exposure had higher adjusted R^2^ than models with disease prevalence as the main exposure (Table 2). For example, adjusted R^2^ was 0.350 for diabetes prevalence but adjusted R^2^ was 0.780 for diabetes hospitalization rate.

When both prevalence of and rate of hospitalization with each chronic condition were entered into the linear regression model, the adjusted R^2^ values increased even more (Table 2). The prevalence of five of the six chronic health conditions (prevalence of cancer remained not statistically significant) and the rate of hospitalization with all six diseases were significantly associated with AKI-D incidence in a positive direction (i.e., higher AKI-D incidence in states with higher chronic health condition prevalence or hospitalization rate). For cancer hospitalization rate, the association was again not linear and more accurately shown graphically in Fig. 3b**.**

With regard to our negative control, we found that skin cancer prevalence and hospitalization rate (Supplementary Table 10) were not correlated with AKI-D incidence (Table 2).

### Sensitivity analysis

In sensitivity analyses using data from 25 states (38,922 AKI-D hospitalizations from the SID (Supplementary Figure 2) and 200,936 observations [weighted population size = 67,096,000] from the BRFSS), we observed similar results in correlation analyses. In regression models, we get similar results expect for cancer prevalence. Cancer prevalence was still a significant factor for AKI-D incidence after several adjustments (coefficient = 154.6, 95% CI 55.6–253.7; *p* < 0.01) (Supplementary Table 11, Supplementary Figure 3).

## Discussion

Epidemiologic studies of kidney disease have focused mostly on ESRD [17] and CKD [18]. Fewer studies have focused on AKI and even fewer on geographic variations in AKI incidence. We recently reported that there is a large difference between states in the incidence of AKI-D [3, 14]. The current study is the first attempt, to our knowledge, to examine factors associated with that variation.

In this national, cross-sectional, ecological study, we found that state-level variation in AKI-D incidence is correlated with state-level variations in prevalence of and state-level variations in hospitalization rate for several chronic health conditions. These findings suggest that geographic distribution of diabetes, hypertension, CKD, ASHD, and COPD (and possibly cancer) may influence geographic distribution of AKI-D.

We acknowledge that firm conclusions cannot be made by ecological studies and correlation is not causation. But, we believe that it is very plausible state-level variations in these chronic health conditions are patho-physiologically relevant since these are known to be risk factors for AKI. For example, diabetes has been recognized as a risk factor for contrast-associated nephropathy and other forms of AKI-D [19, 20]. Hypertension, common among adults, has been linked to AKI [21, 22]. CKD is closely related to AKI and severity of CKD is associated with progressively higher risk of AKI [23, 24]. AKI is common in ASHD patients who have undergone coronary artery bypass graft surgery [25] or percutaneous coronary intervention [26]. COPD patients have increased incidence of bacteremia, likely reflecting greater vulnerability to acquiring severe infections [27, 28], and this higher prevalence of respiratory tract infection-related septicemia could increase the number of AKI-D episodes. AKI-D can be cancer-related due to intrinsic renal factors, postrenal factors and chemotherapeutic agents, which may be intensified in the inpatient setting.

We believe our conclusions are strengthened by the findings regarding skin cancer, our negative control (which was also selected a priori). We had hypothesized that state-level variation in skin cancer prevalence and hospitalization rate should not correlate or be associated with state-level AKI-D since skin-cancer is not a known AKI risk factor. We indeed observed this. Thus, this provides some reassurance about the specificity of the observed state-level associations between the 6 selected chronic health conditions and AKI-D.

We believe that a potentially important observation is that state-level differences in rates of hospitalization with chronic health conditions (e.g., up to 3.2-fold difference in range) were larger than the state-level difference in prevalence for each chronic health condition (e.g., up to 2.5-fold difference in range). Furthermore, as noted, for most of the chronic conditions, the adjusted R^2^ was higher in the models with hospitalization rate as the main exposure vs. the models with disease prevalence as the main exposure, which is consistent with the hypothesis that rates of hospitalization with chronic health conditions are more influential than prevalence rates in the community in explaining state-level variation in AKI-D incidence. Since it is plausible that patients with poorly controlled chronic health conditions are the ones more likely to be admitted, these results highlight the possibility that improved management of chronic health conditions could reduce AKI-D episodes. Our study potentially identify which states would garner the most benefit in terms of these prevention strategies. Overall, intensive patient education programs to improve self-management of chronic conditions and the skill and supply of non-nephrologists (such as endocrinologists, cardiologists, pulmonologists, and primary physicians) may be important modifiable factors to reduce AKI-D disease burden.

Our study is strengthened by encompassing multiple states across the nation. To our knowledge, the BRFSS is the only comprehensive state-level dataset available regarding the chronic health conditions of interest. Previous studies reported that BRFSS data highly agree with data from other national surveys [29–31]. As a major improvement of the 2011 BRFSS, the inclusion of survey data from participants using cellular telephones in addition to data collected via landline telephones increased representativeness and accuracy [32]. With regard to defining AKI-D incidence, we captured AKI-D cases using the best-validated set of ICD-9-CM diagnostic and procedure codes [3, 12–14].

However, we recognize a number of limitations. Due to limitations in the SID data, we did not have data on all 50 states. Thus our results may not generalize to the entire country. We lacked clinical data (such as creatinine or blood glucose levels, physician documentation of medical history or physical examination such as body mass index, medications prescribed) to capture AKI-D and chronic health conditions and this may introduce biases in our estimations of the strengths of associations. We identified each health condition using diagnostic codes and so were not able to capture disease severity. We were unable to quantify any pre-existing reductions in glomerular filtration rate for those patients who developed AKI-D. There were other factors which may contribute to AKI-D incidence (such as state-level variation in economic status, medical and social services, mix of academic vs. non-academic hospital, number of intensive care unit bed) which we did not have available information to adjust for. We were not able to account for any potential differences in threshold to initiate dialysis or death at home as a competing event that may vary regionally. Likely because of reliance on patient self-report, the population prevalence of CKD according to BRFSS is much lower than the population prevalence of CKD defined using biochemical markers from the National Health and Nutrition Examination Survey (NHANES) [33, 34]. This misclassification may also explain why we did not see stronger correlations between AKI-D incidence and CKD prevalence or AKI-D incidence and rate of hospitalization with CKD in unadjusted analyses, despite CKD being a very strong AKI risk factor. In addition, BRFSS does not include those who live in nursing homes or other facilities and relies on respondent being physically and mentally able to respond to the survey. Thus people with severe comorbidities are under-represented. The cross-sectional and ecological study design meant we did not have individual level data to make stronger inferences.

## Conclusion

There is considerable state-level variation in the incidence of dialysis-requiring acute kidney injury (AKI-D). AKI-D incidence was associated with prevalence of and rates of hospitalization with five chronic health conditions--diabetes, hypertension, CKD, ASHD and COPD--and rates of hospitalization with cancer. We believe our results are provocative and shed light on potential reasons for the geographic variations in AKI-D incidence. Our results, if replicated by others, suggest that measures which reduce hospitalization of patients with chronic health conditions may translate into lower incidence of AKI-D.

## Supplementary information

**Additional file 1 Supplementary Table 1** The BRFSS questionnaires included in our study. **Supplementary Table 2** State-level AKI-D incidence in 2011 for the 18 states in the primary analysis. **Supplementary Table 3** State-level demographic characteristics of BRFSS respondents in 2011 for the 18 states in the primary analysis. **Supplementary Table 4** State-level prevalence estimates for diabetes mellitus and rates of hospitalization with diabetes mellitus in 2011 for the 18 states in the primary analysis **Supplementary Table 5** State-level prevalence estimates for hypertension and rates of hospitalization with hypertension in 2011 for the 18 states in the primary analysis. **Supplementary Table 6** State-level prevalence estimates for chronic kidney disease and rates of hospitalization with chronic kidney disease in 2011 for the 18 states in the primary analysis. **Supplementary Table 7** State-level prevalence estimates for arteriosclerotic heart disease and rates of hospitalization with arteriosclerotic heart disease in 2011 for the 18 states in the primary analysis. **Supplementary Table 8** State-level prevalence estimates for cancer and rates of hospitalization with cancer in 2011 for the 18 states in the primary analysis. **Supplementary Table 9** State-level prevalence estimates for chronic obstructive pulmonary disease and rates of hospitalization with chronic obstructive pulmonary disease in 2011 for the 18 states in the primary analysis. **Supplementary Table 10** State-level prevalence estimates for skin cancer and rates of hospitalization with skin cancer in 2011 for the 18 states in the primary analysis. **Supplementary Table 11** Linear regression model for AKI-D incidence and each chronic health condition for the 25 states in the sensitivity analysis. **Supplementary Figure 1** Flow diagram showing selection of state for data analysis. **Supplementary Figure 2** Map demonstrating the regional variation of AKI-D incidence in sensitivity analysis with 25 states. **Supplementary Figure 3** Rate of hospitalization with cancer and predicted AKI-D incidence for the 25 states in the sensitivity analysis. A) Adjusted for age, % male, and % non-Hispanic White. B) Adjusted for age, % male, % non-Hispanic White, and prevalence of cancer.

## Data Availability

The public access to BRFSS data is open (https://www.cdc.gov/brfss/index.html). The SID databases can be purchased from the Healthcare Cost and Utilization Project (HCUP) (https://www.hcup-us.ahrq.gov/home.jsp). The US Census data are available from https://www.census.gov .

## Abbreviations

AKI-D: Dialysis-requiring acute kidney injury
SID: State Inpatient Databases
BRFSS: The Behavioral Risk Factor Surveillance System
CKD: Chronic kidney disease
ASHD: Arteriosclerotic heart disease
COPD: Chronic obstructive pulmonary disease
ESRD: End-stage renal disease
USRDS: The United States Renal Data System
CVA/TIA: Cerebrovascular accident/transient ischemic attack
CSS: Clinical Classifications Software
ICD-9-CM: The International Classification of Diseases-Ninth Revision-Clinical Modification
pmp: Per million population
CIs: Confidence intervals
