# Supplementary material for: Exploring reasons for state-level variation in incidence of dialysis-requiring acute kidney injury (AKI-D) in the United States
Source: BMC Nephrol. 2020 Aug 10;21:336. doi: 10.1186/s12882-020-02000-7 (PMC7418406; doi:10.1186/s12882-020-02000-7)
Supplement: Supplementary file 1 — Additional file 1 Supplementary Table 1 The BRFSS questionnaires included in our study. Supplementary Table 2 State-level AKI-D incidence in 2011 for the 18 states in the primary analysis. Supplementary Table 3 State-level demographic characteristics of BRFSS respondents in 2011 for the 18 states in the primary analysis. Supplementary Table 4 State-level prevalence estimates for diabetes mellitus and rates of hospitalization with diabetes mellitus in 2011 for the 18 states in the primary analysis Supplementary Table 5 State-level prevalence estimates for hypertension and rates of hospitalization with hypertension in 2011 for the 18 states in the primary analysis. Supplementary Table 6 State-level prevalence estimates for chronic kidney disease and rates of hospitalization with chronic kidney disease in 2011 for the 18 states in the primary analysis. Supplementary Table 7 State-level prevalence estimates for arteriosclerotic heart disease and rates of hospitalization with arteriosclerotic heart disease in 2011 for the 18 states in the primary analysis. Supplementary Table 8 State-level prevalence estimates for cancer and rates of hospitalization with cancer in 2011 for the 18 states in the primary analysis. Supplementary Table 9 State-level prevalence estimates for chronic obstructive pulmonary disease and rates of hospitalization with chronic obstructive pulmonary disease in 2011 for the 18 states in the primary analysis. Supplementary Table 10 State-level prevalence estimates for skin cancer and rates of hospitalization with skin cancer in 2011 for the 18 states in the primary analysis. Supplementary Table 11 Linear regression model for AKI-D incidence and each chronic health condition for the 25 states in the sensitivity analysis. Supplementary Figure 1 Flow diagram showing selection of state for data analysis. Supplementary Figure 2 Map demonstrating the regional variation of AKI-D incidence in sensitivity analysis with 25 states. Supplementary Figure 3 Rate of h [file 12882_2020_2000_MOESM1_ESM.docx]

**Supplementary Table 1** The BRFSS questionnaires included in our study

| Category | Questions | Answers |
| --- | --- | --- |
| Diabetes | “(Ever told) you have diabetes?” | “Yes”, “Yes, but female told only during pregnancy”, “No”, “No, pre-diabetes or borderline diabetes”, “Don’t know/not sure”, “Refused” |
| Hypertension | “Have you EVER been told by a doctor, nurse, or other health professional that you have high blood pressure?” | “Yes”, “Yes, but female told only during pregnancy”, “No”, “Told borderline high or pre-hypertensive”, “Don’t know/not sure”, “Refused” |
| Chronic Kidney Disease | “(Ever told) you have kidney disease? Do NOT include kidney stones, bladder infections or incontinence.” | “Yes”, “No”, “Don’t know/not sure”, “Refused” |
| Arteriosclerotic Heart Disease | “(Ever told) you that you had a heart attack, also called a myocardial infarction? ” | “Yes”, “No”, “Don’t know/not sure”, “Refused” |
|  | “(Ever told) you had angina or coronary heart disease? ” | “Yes”, “No”, “Don’t know/not sure”, “Refused” |
| Cancer  (excluding skin cancer) | “(Ever told) you had any other type of cancer?” (Not skin cancer) | “Yes”, “No”, “Don’t know/not sure”, “Refused” |
| Chronic Obstructive Pulmonary Disease | “(Ever told) you have chronic obstructive pulmonary disease(COPD), emphysema or chronic bronchitis?” | “Yes”, “No”, “Don’t know/not sure”, “Refused” |
| Skin Cancer | “(Ever told) you had skin cancer?” | “Yes”, “No”, “Don’t know/not sure”, “Refused” |

**Supplementary Table 2** State-level AKI-D incidence in 2011 for the 18 states in the primary analysis

| State | Population | Number of AKI-D hospitalizations | AKI-D incidence  (pmp) | Mean Age (years) | Proportion of Male | Proportion of Non-Hispanic White |
| --- | --- | --- | --- | --- | --- | --- |
| Arizona | 2,515,782 | 1838 | 730 | 67.1 | 0.59 | 0.76 |
| Arkansas | 1,193,793 | 850 | 712 | 67.4 | 0.58 | 0.82 |
| California | 13,874,828 | 8196 | 590 | 68.4 | 0.58 | 0.55 |
| Florida | 8,556,718 | 5504 | 643 | 69.3 | 0.59 | 0.70 |
| Iowa | 1,277,295 | 596 | 467 | 68.1 | 0.61 | 0.94 |
| Kentucky | 1,787,705 | 1393 | 779 | 66.7 | 0.55 | 0.90 |
| Maryland | 2,360,002 | 1417 | 600 | 67.0 | 0.56 | 0.60 |
| Massachusetts | 2,773,244 | 1304 | 470 | 68.5 | 0.60 | 0.85 |
| Michigan | 4,177,100 | 2808 | 672 | 67.7 | 0.57 | 0.76 |
| Nevada | 1,042,774 | 1188 | 1139 | 67.5 | 0.60 | 0.71 |
| New Jersey | 3,677,622 | 1938 | 527 | 69.0 | 0.57 | 0.68 |
| New Mexico | 833,432 | 287 | 344 | 66.7 | 0.56 | 0.48 |
| New York | 7,939,019 | 3957 | 498 | 68.9 | 0.56 | 0.62 |
| Oregon | 1,608,913 | 598 | 372 | 66.1 | 0.58 | 0.87 |
| Rhode Island | 449,774 | 175 | 389 | 68.1 | 0.55 | 0.89 |
| South Carolina* | 1,922,696 | 918 | 477 | NA | 0.56 | 0.63 |
| Vermont | 287,291 | 55 | 191 | 66.8 | 0.64 | 0.94 |
| Washington | 2,709,335 | 1100 | 406 | 66.3 | 0.58 | 0.86 |

** SC reported age only as categories*

**Supplementary Table 3** State-level demographic characteristics of BRFSS respondents in 2011 for the 18 states in the primary analysis

| State | Mean Age (years) | Proportion of Male | Proportion of Non-Hispanic White |
| --- | --- | --- | --- |
| Arizona | 61.5 | 0.48 | 0.70 |
| Arkansas | 61.8 | 0.47 | 0.81 |
| California | 60.4 | 0.47 | 0.55 |
| Florida | 62.8 | 0.47 | 0.70 |
| Iowa | 61.8 | 0.47 | 0.94 |
| Kentucky | 61.1 | 0.47 | 0.89 |
| Maryland | 60.4 | 0.46 | 0.64 |
| Massachusetts | 61.1 | 0.46 | 0.84 |
| Michigan | 61.0 | 0.47 | 0.82 |
| Nevada | 60.5 | 0.50 | 0.67 |
| New Jersey | 60.9 | 0.47 | 0.69 |
| New Mexico | 61.2 | 0.47 | 0.52 |
| New York | 61.2 | 0.46 | 0.67 |
| Oregon | 61.3 | 0.48 | 0.87 |
| Rhode Island | 61.5 | 0.46 | 0.86 |
| South Carolina* | 61.3 | 0.46 | 0.72 |
| Vermont | 61.1 | 0.48 | 0.96 |
| Washington | 60.7 | 0.48 | 0.82 |

**Supplementary Table 4** State-level prevalence estimates for diabetes mellitus and rates of hospitalization with diabetes mellitus in 2011 for the 18 states in the primary analysis

| State | Estimated Prevalence (%) | 95% CI | | Unweighted Cases | Unweighted Sample Size | Hospitalization Rate  (per 1,000 patients) | |
| --- | --- | --- | --- | --- | --- | --- | --- |
|  |  | **Low** | **Upper** |  |  |  |  |
| Arizona | 15.7 | 13.8 | 17.7 | 745 | 4,952 | 354.4 |  |
| Arkansas | 17.6 | 16.0 | 19.2 | 692 | 3,767 | 374.4 |  |
| California | 14.3 | 13.4 | 15.2 | 1,689 | 12,308 | 344.8 |  |
| Florida | 16.5 | 15.4 | 17.7 | 1,670 | 9,430 | 375.9 |  |
| Iowa | 13.9 | 12.8 | 15.0 | 749 | 5,216 | 367.9 |  |
| Kentucky | 17.5 | 16.3 | 18.7 | 1,611 | 8,099 | 438.0 |  |
| Maryland | 15.3 | 14.1 | 16.6 | 1,109 | 7,160 | 396.7 |  |
| Massachusetts | 12.8 | 12.0 | 13.6 | 2,335 | 15,773 | 423.5 |  |
| Michigan | 15.6 | 14.6 | 16.8 | 1,364 | 8,081 | 403.0 |  |
| Nevada | 17.1 | 14.8 | 19.6 | 520 | 3,831 | 278.9 |  |
| New Jersey | 14.2 | 13.2 | 15.2 | 1,598 | 10,727 | 381.4 |  |
| New Mexico | 16.2 | 15.2 | 17.3 | 1,110 | 6,764 | 251.9 |  |
| New York | 17.0 | 15.7 | 18.4 | 798 | 5,000 | 350.2 |  |
| Oregon | 15.0 | 13.7 | 16.5 | 655 | 4,553 | 266.5 |  |
| Rhode Island | 13.3 | 12.1 | 14.5 | 625 | 4,722 | 392.5 |  |
| South Carolina | 18.4 | 17.3 | 19.7 | 1,886 | 9,421 | 321.2 |  |
| Vermont | 11.9 | 10.9 | 13.0 | 658 | 5,386 | 269.9 |  |
| Washington | 14.5 | 13.5 | 15.6 | 1,610 | 11,302 | 267.9 |  |

**Supplementary Table 5** State-level prevalence estimates for hypertension and rates of hospitalization with hypertension in 2011 for the 18 states in the primary analysis

| State | Estimated Prevalence (%) | 95% CI | | Unweighted Cases | Unweighted Sample Size | Hospitalization Rate  (per 1,000 patients) |
| --- | --- | --- | --- | --- | --- | --- |
|  |  | **Low** | **Upper** |  |  |  |
| Arizona | 42.5 | 40.1 | 45.0 | 2,383 | 4,952 | 264.1 |
| Arkansas | 54.1 | 51.9 | 56.3 | 2,125 | 3,767 | 247.1 |
| California | 44.8 | 43.6 | 46.1 | 5,731 | 12,308 | 213.4 |
| Florida | 49.6 | 48.0 | 51.1 | 4,988 | 9,430 | 267.6 |
| Iowa | 44.3 | 42.7 | 46.0 | 2,482 | 5,216 | 229.7 |
| Kentucky | 53.9 | 52.2 | 55.6 | 4,698 | 8,099 | 273.1 |
| Maryland | 46.9 | 45.2 | 48.5 | 3,552 | 7,160 | 263.0 |
| Massachusetts | 43.1 | 41.9 | 44.4 | 7,249 | 15,773 | 278.4 |
| Michigan | 49.9 | 48.3 | 51.5 | 4,173 | 8,081 | 266.9 |
| Nevada | 46.2 | 43.3 | 49.1 | 1,801 | 3,831 | 223.5 |
| New Jersey | 45.2 | 43.9 | 46.6 | 5,066 | 10,727 | 254.3 |
| New Mexico | 44.2 | 42.7 | 45.7 | 3,136 | 6,764 | 170.9 |
| New York | 46.6 | 44.8 | 48.3 | 2,375 | 5,000 | 268.2 |
| Oregon | 43.7 | 41.9 | 45.6 | 2,084 | 4,553 | 179.4 |
| Rhode Island | 48.9 | 47.2 | 50.7 | 2,320 | 4,722 | 243.7 |
| South Carolina | 53.8 | 52.3 | 55.4 | 5,358 | 9,421 | 216.0 |
| Vermont | 42.1 | 40.5 | 43.7 | 2,359 | 5,386 | 153.0 |
| Washington | 45.0 | 43.7 | 46.4 | 5,354 | 11,302 | 173.1 |

**Supplementary Table 6** State-level prevalence estimates for chronic kidney disease and rates of hospitalization with chronic kidney disease in 2011 for the 18 states in the primary analysis

| State | Estimated Prevalence (%) | 95% CI | | Unweighted Cases | Unweighted Sample Size | Hospitalization Rate  (per 1,000 patients) |
| --- | --- | --- | --- | --- | --- | --- |
|  |  | **Low** | **Upper** |  |  |  |
| Arizona | 5.9 | 4.8 | 7.4 | 262 | 4,952 | 433.1 |
| Arkansas | 4.6 | 3.7 | 5.6 | 168 | 3,767 | 615.4 |
| California | 3.9 | 3.5 | 4.5 | 564 | 12,308 | 678.6 |
| Florida | 4.3 | 3.7 | 5.0 | 445 | 9,430 | 696.0 |
| Iowa | 2.4 | 1.9 | 2.9 | 131 | 5,216 | 1040.0 |
| Kentucky | 3.5 | 2.9 | 4.1 | 321 | 8,099 | 983.7 |
| Maryland | 2.8 | 2.3 | 3.4 | 217 | 7,160 | 1144.2 |
| Massachusetts | 2.7 | 2.3 | 3.1 | 432 | 15,773 | 1118.3 |
| Michigan | 4.1 | 3.5 | 4.7 | 395 | 8,081 | 848.3 |
| Nevada | 4.0 | 3.2 | 5.2 | 172 | 3,831 | 536.6 |
| New Jersey | 2.6 | 2.2 | 3.0 | 313 | 10,727 | 1072.0 |
| New Mexico | 4.8 | 4.2 | 5.5 | 321 | 6,764 | 360.9 |
| New York | 3.7 | 3.1 | 4.4 | 194 | 5,000 | 770.9 |
| Oregon | 3.4 | 2.9 | 4.1 | 165 | 4,553 | 583.8 |
| Rhode Island | 3.4 | 2.9 | 4.1 | 174 | 4,722 | 743.9 |
| South Carolina | 3.5 | 3.0 | 4.1 | 388 | 9,421 | 828.0 |
| Vermont | 3.1 | 2.6 | 3.7 | 189 | 5,386 | 471.9 |
| Washington | 3.1 | 2.7 | 3.6 | 389 | 11,302 | 673.5 |

**Supplementary Table 7** State-level prevalence estimates for arteriosclerotic heart disease and rates of hospitalization with arteriosclerotic heart disease in 2011 for the 18 states in the primary analysis

| State | Estimated Prevalence (%) | 95% CI | | Unweighted Cases | Unweighted Sample Size | Hospitalization Rate  (per 1,000 patients) |
| --- | --- | --- | --- | --- | --- | --- |
|  |  | **Low** | **Upper** |  |  |  |
| Arizona | 11.3 | 9.9 | 12.9 | 664 | 4,952 | 437.0 |
| Arkansas | 15.8 | 14.3 | 17.4 | 612 | 3,767 | 442.1 |
| California | 9.6 | 8.9 | 10.3 | 1,294 | 12,308 | 392.0 |
| Florida | 13.3 | 12.3 | 14.3 | 1,368 | 9,430 | 459.5 |
| Iowa | 10.0 | 9.1 | 11.0 | 565 | 5,216 | 479.3 |
| Kentucky | 14.9 | 13.8 | 16.2 | 1,269 | 8,099 | 523.0 |
| Maryland | 10.5 | 9.5 | 11.6 | 775 | 7,160 | 484.5 |
| Massachusetts | 9.8 | 9.1 | 10.6 | 1,688 | 15,773 | 543.4 |
| Michigan | 13.1 | 12.0 | 14.3 | 1,077 | 8,081 | 495.2 |
| Nevada | 12.1 | 10.5 | 14.0 | 471 | 3,831 | 347.4 |
| New Jersey | 10.9 | 10.1 | 11.7 | 1,205 | 10,727 | 512.4 |
| New Mexico | 10.6 | 9.7 | 11.5 | 760 | 6,764 | 288.9 |
| New York | 10.4 | 9.4 | 11.5 | 539 | 5,000 | 546.6 |
| Oregon | 9.3 | 8.3 | 10.3 | 464 | 4,553 | 367.1 |
| Rhode Island | 11.0 | 9.9 | 12.1 | 524 | 4,722 | 499.7 |
| South Carolina | 12.3 | 11.4 | 13.4 | 1,203 | 9,421 | 415.6 |
| Vermont | 10.0 | 9.1 | 11.1 | 554 | 5,386 | 316.0 |
| Washington | 9.7 | 8.9 | 10.5 | 1,148 | 11,302 | 338.3 |

**Supplementary Table 8** State-level prevalence estimates for cancer and rates of hospitalization with cancer in 2011 for the 18 states in the primary analysis

| State | Estimated Prevalence (%) | 95% CI | | Unweighted Cases | Unweighted Sample Size | Hospitalization Rate  (per 1,000 patients) |
| --- | --- | --- | --- | --- | --- | --- |
|  |  | **Low** | **Upper** |  |  |  |
| Arizona | 11.4 | 10.1 | 12.9 | 662 | 4,952 | 265.1 |
| Arkansas | 10.7 | 9.5 | 12.1 | 472 | 3,767 | 303.8 |
| California | 9.5 | 8.9 | 10.2 | 1,524 | 12,308 | 273.5 |
| Florida | 11.9 | 11.0 | 12.9 | 1,346 | 9,430 | 290.3 |
| Iowa | 9.8 | 8.9 | 10.8 | 583 | 5,216 | 296.8 |
| Kentucky | 10.7 | 9.7 | 11.7 | 1,016 | 8,099 | 326.8 |
| Maryland | 10.3 | 9.4 | 11.4 | 794 | 7,160 | 325.5 |
| Massachusetts | 10.7 | 10.0 | 11.5 | 1,890 | 15,773 | 384.2 |
| Michigan | 11.5 | 10.6 | 12.5 | 1,067 | 8,081 | 304.9 |
| Nevada | 10.4 | 8.8 | 12.2 | 472 | 3,831 | 249.5 |
| New Jersey | 9.1 | 8.4 | 9.9 | 1,144 | 10,727 | 359.0 |
| New Mexico | 9.6 | 8.8 | 10.5 | 760 | 6,764 | 197.5 |
| New York | 11.2 | 10.1 | 12.3 | 629 | 5,000 | 339.8 |
| Oregon | 11.7 | 10.5 | 12.9 | 624 | 4,553 | 222.8 |
| Rhode Island | 11.7 | 10.6 | 12.8 | 597 | 4,722 | 304.4 |
| South Carolina | 10.4 | 9.5 | 11.3 | 1,109 | 9,421 | 273.0 |
| Vermont | 10.5 | 9.6 | 11.5 | 632 | 5,386 | 204.2 |
| Washington | 10.8 | 10.1 | 11.6 | 1,502 | 11,302 | 234.9 |

**Supplementary Table 9** State-level prevalence estimates for chronic obstructive pulmonary disease and rates of hospitalization with chronic obstructive pulmonary disease in 2011 for the 18 states in the primary analysis

| State | Estimated Prevalence (%) | 95% CI | | Unweighted Cases | Unweighted Sample Size | Hospitalization Rate  (per 1,000 patients) |
| --- | --- | --- | --- | --- | --- | --- |
|  |  | **Low** | **Upper** |  |  |  |
| Arizona | 8.6 | 7.5 | 9.9 | 537 | 4,952 | 343.7 |
| Arkansas | 12.6 | 11.3 | 14.1 | 490 | 3,767 | 340.8 |
| California | 6.4 | 5.9 | 7.0 | 980 | 12,308 | 352.4 |
| Florida | 10.9 | 10.0 | 11.8 | 1,233 | 9,430 | 356.1 |
| Iowa | 7.4 | 6.6 | 8.4 | 401 | 5,216 | 465.0 |
| Kentucky | 13.5 | 12.4 | 14.7 | 1,214 | 8,099 | 473.5 |
| Maryland | 8.0 | 7.2 | 9.0 | 595 | 7,160 | 394.6 |
| Massachusetts | 8.5 | 7.8 | 9.2 | 1,536 | 15,773 | 393.4 |
| Michigan | 11.5 | 10.5 | 12.6 | 933 | 8,081 | 361.2 |
| Nevada | 11.5 | 9.7 | 13.7 | 424 | 3,831 | 278.4 |
| New Jersey | 7.3 | 6.6 | 8.0 | 867 | 10,727 | 406.4 |
| New Mexico | 9.6 | 8.7 | 10.6 | 664 | 6,764 | 212.9 |
| New York | 8.3 | 7.4 | 9.3 | 431 | 5,000 | 340.8 |
| Oregon | 8.8 | 7.8 | 10.0 | 413 | 4,553 | 257.1 |
| Rhode Island | 9.4 | 8.4 | 10.5 | 448 | 4,722 | 378.6 |
| South Carolina | 11.0 | 10.1 | 11.9 | 1,073 | 9,421 | 301.3 |
| Vermont | 7.6 | 6.7 | 8.5 | 428 | 5,386 | 307.9 |
| Washington | 6.3 | 5.7 | 7.1 | 786 | 11,302 | 360.9 |

**Supplementary Table 10** State-level prevalence estimates for skin cancer and rates of hospitalization with skin cancer in 2011 for the 18 states in the primary analysis

| State | Estimated Prevalence (%) | 95% CI | | Unweighted Cases | Unweighted Sample Size | Hospitalization Rate  (per 1,000 patients) |
| --- | --- | --- | --- | --- | --- | --- |
|  |  | **Low** | **Upper** |  |  |  |
| Arizona | 12.9 | 11.5 | 14.4 | 749 | 4,952 | 23.7 |
| Arkansas | 10.9 | 9.8 | 12.2 | 460 | 3,767 | 18.3 |
| California | 9.7 | 9.1 | 10.3 | 1,795 | 12,308 | 21.8 |
| Florida | 14.7 | 13.8 | 15.6 | 1,328 | 9,430 | 18.2 |
| Iowa | 9.5 | 8.7 | 10.5 | 1,844 | 5,216 | 19.5 |
| Kentucky | 11.5 | 10.5 | 12.5 | 559 | 8,099 | 25.0 |
| Maryland | 7.8 | 7.0 | 8.5 | 940 | 7,160 | 30.3 |
| Massachusetts | 9.2 | 8.5 | 9.9 | 1,070 | 15,773 | 39.1 |
| Michigan | 9.8 | 8.9 | 10.7 | 738 | 8,081 | 26.6 |
| Nevada | 9.3 | 7.8 | 11.0 | 1,507 | 3,831 | 15.7 |
| New Jersey | 8.1 | 7.5 | 8.8 | 854 | 10,727 | 24.1 |
| New Mexico | 10.0 | 9.2 | 10.8 | 778 | 6,764 | 13.7 |
| New York | 7.7 | 6.9 | 8.5 | 2,044 | 5,000 | 29.8 |
| Oregon | 11.1 | 10.1 | 12.1 | 505 | 4,553 | 20.9 |
| Rhode Island | 10.5 | 9.5 | 11.5 | 1,081 | 4,722 | 17.2 |
| South Carolina | 11.5 | 10.7 | 12.4 | 807 | 9,421 | 18.8 |
| Vermont | 9.4 | 8.5 | 10.3 | 489 | 5,386 | 23.2 |
| Washington | 10.1 | 9.4 | 10.9 | 1,142 | 11,302 | 20.7 |

**Supplementary Table 11** Linear regression model for AKI-D incidence and each chronic health condition for the 25 states in the sensitivity analysis

|  | Prevalence （%） | | Hospitalization Rate  (per 1,000 patients) | |  |
| --- | --- | --- | --- | --- | --- |
|  | Coef.（95% CI） | P value | Coef.（95% CI） | P value | Adjusted R^2^ |
| *Model 1* |  |  |  |  |  |
| DM | 50.2 (13.2,87.1) | 0.01 | - | - | 0.248 |
| HTN | 27.9 (8.7,47.1) | <0.01 | - | - | 0.277 |
| CKD | 58.5 (-64.9,181.8) | 0.33 | - | - | 0.187 |
| ASHD | 69.1 (34.3,103.8） | <0.01 | - | - | 0.434 |
| Cancer (excluding skin cancer) | 28.6 (-93.6,150.8) | 0.63 | - | - | 0.155 |
| COPD | 49.6 (13.8,85.4) | <0.01 | - | - | 0.257 |
| Skin Cancer | 0.8 (-51.7,53.3) | 0.98 |  |  | 0.145 |
| *Model 2* |  |  |  |  |  |
| DM | - | - | 3.6 (2.5,4.8) | <0.01 | 0.740 |
| HTN | - | - | 4.7 (3.1,6.3) | <0.01 | 0.708 |
| CKD | - | - | 0.6 (0.1,1.1) | 0.02 | 0.365 |
| ASHD | - | - | 2.1 (0.8,3.3) | <0.01 | 0.484 |
| Cancer (excluding skin cancer) | - | - | See Supplementary Figure 3A | <0.01 | 0.598 |
| COPD | - | - | 2.2 (1.2,3.3) | <0.01 | 0.586 |
| Skin Cancer |  |  | 16.0 (-7.4,39.4) | 0.17 | 0.228 |
| *Model 3* |  |  |  |  |  |
| DM | 37.8 (20.3,55.2) | <0.001 | 3.6 (2.8,4.4) | <0.01 | 0.872 |
| HTN | 20.2 (10.1,30.3) | 0.001 | 4.7 (3.4,5.9) | <0.01 | 0.811 |
| CKD | 220.7 (131.1,310.3) | <0.001 | 1.2 (0.8,1.6) | <0.01 | 0.731 |
| ASHD | 54.6 (30.3,78.9) | <0.001 | 1.9 (1.0,2.7) | <0.01 | 0.757 |
| Cancer (excluding skin cancer) | 57.0 (-38.8,152.8) | 0.227 | See Supplementary Figure 3B | <0.01 | 0.554 |
| COPD | 30.4 (2.9,57.9) | 0.032 | 2.1 (1.2,3.0) | <0.01 | 0.663 |
| Skin Cancer | 21.1 (-36.0,78.3) | 0.447 | 20.6 (-6.2,47.4) | 0.12 | 0.211 |
| Model 1: adjusted for mean age, % male, % non-Hispanic White, prevalence of each chronic health condition  Model 2: adjusted for mean age, % male, % non-Hispanic White, hospitalization rate of each chronic health condition  Model 3: adjusted for mean age, % male, % non-Hispanic White, prevalence of each chronic health condition and hospitalization rate of each chronic health condition | | | | | |

Abbrevations: ASHD, arteriosclerotic heart disease ; CKD, chronic kidney disease; COPD, chronic obstructive pulmonary disease.

**Supplementary Figure 1** Flow diagram showing selection of state for data analysis

**Supplementary Figure 2** Map demonstrating the regional variation of AKI-D incidence in sensitivity analysis with 25 states


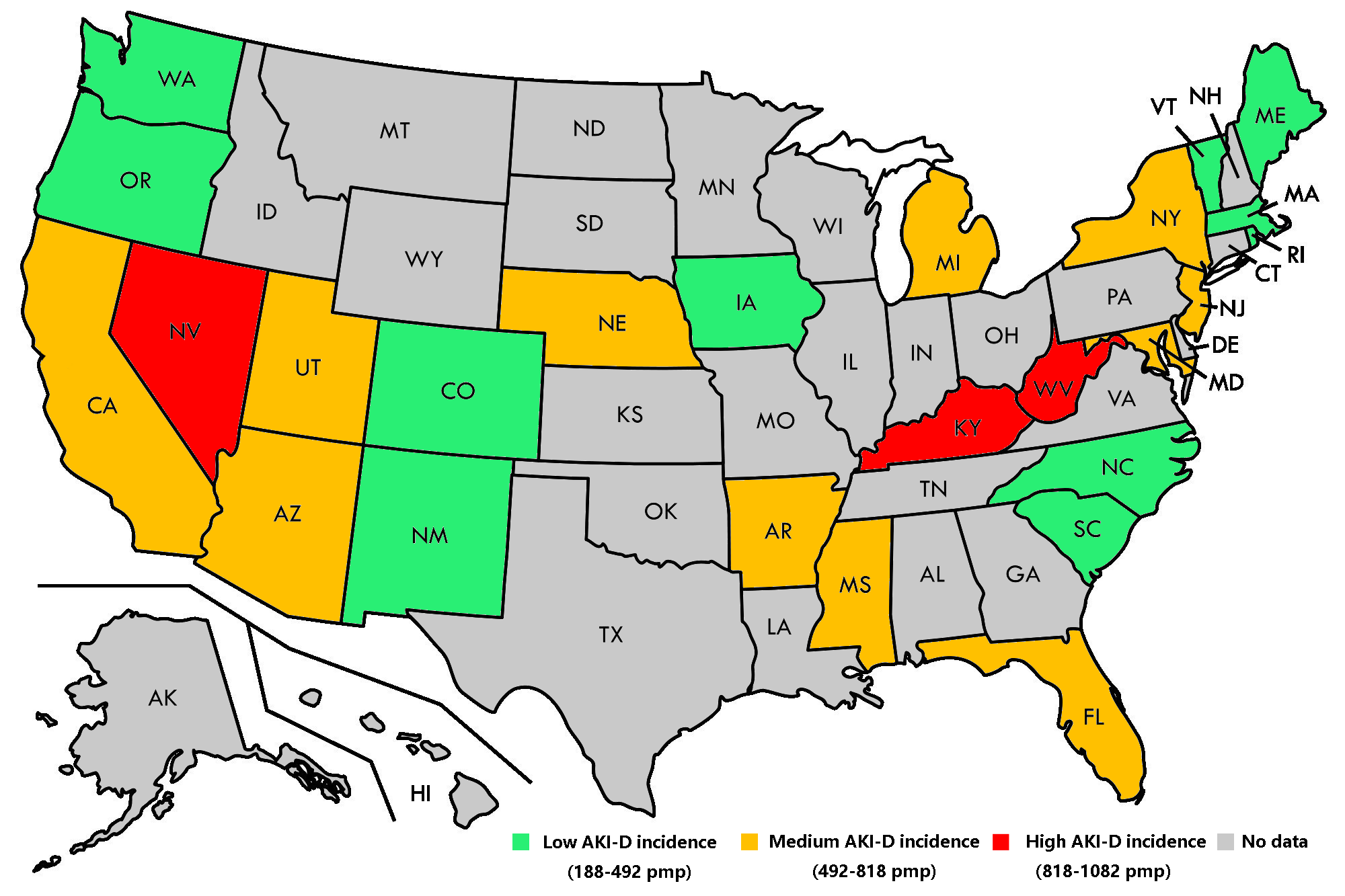


The map was drawn using Adobe Photoshop CS6 version 13.1.2 URL:http://www.adobe.com

**Supplementary Figure 3** Rate of hospitalization with cancer and predicted AKI-D incidence for the 25 states in the sensitivity analysis.

A) Adjusted for age, % male, and % non-Hispanic White.

B) Adjusted for age, % male, % non-Hispanic White, and prevalence of cancer.

A)

B)
